# Medial joint space narrowing progresses after pullout repair of medial meniscus posterior root tear

**DOI:** 10.1007/s00264-023-05701-4

**Published:** 2023-01-30

**Authors:** Koki Kawada, Takayuki Furumatsu, Masanori Tamura, Haowei Xue, Naohiro Higashihara, Keisuke Kintaka, Yusuke Yokoyama, Toshifumi Ozaki

**Affiliations:** 1 Department of Orthopaedic Surgery, Okayama University Graduate School of Medicine, Dentistry, and Pharmaceutical Science, Okayama, Japan; 2 Present Address: Present address: Department of Orthopaedic Surgery, Okayama University Hospital, 2-5-1 Shikata-cho, Kita-ku, Okayama, 700-8558 Japan

**Keywords:** Fixed-flexion view, Medial joint space, Medial meniscus extrusion, Meniscus, Posterior root tear, Pullout repair

## Abstract

**Purpose:**

The extent to which arthropathic changes progress after medial meniscus posterior root tear (MMPRT) repair remains controversial. This retrospective study assessed medial joint space (MJS) narrowing progression after pullout repair for MMPRT and identified the correlating factors.

**Methods:**

We included 56 patients who underwent pullout repair for MMPRT. The MJS of the bilateral knees was assessed with radiography using the fixed-flexion view. A second-look arthroscopy was performed one year post-operatively for all patients. The baseline characteristics, clinical scores, Kellgren–Lawrence (KL) grade, and medial meniscus extrusion (MME) were identified. Statistical comparisons and correlation analyses were conducted.

**Results:**

The MJS narrowing width was significantly larger in MMPRT knees than in contralateral knees (0.51 ± 0.85 mm vs. 0.09 ± 0.49 mm, *p* < 0.001). KL grade progression was observed in 23.2% (13/56) of patients. There was a significant difference between pre- and post-operative MME values, indicating MME progression (*p* < 0.001). Each clinical score showed significant improvement one year post-operatively (*p* < 0.001). Positive correlations were found between MJS narrowing and pre-operative MJS (coefficient = 0.510, *p* < 0.001), rate of change in MJS (coefficient = 0.929, *p* < 0.001), and increase in MME (ΔMME) (coefficient = 0.506, *p* < 0.001).

**Conclusion:**

Knees that underwent pullout repair for MMPRT showed progression of MJS narrowing by 0.51 mm at one year post-operatively, although clinical scores markedly improved. Correlating factors for MJS narrowing were pre-operative MJS, rate of change in MJS, and ΔMME. Preventing MME progression is essential for preventing arthropathic changes.

## Introduction

The posterior root of the medial meniscus (MM) anchors the meniscus to the bone. An MM posterior root tear (MMPRT) interferes with the conversion of axial stress into hoop tension and adversely affects load transfer through the meniscus [1].

Historically, the treatment options for MMPRT have been either conservative or partial meniscectomy. However, several studies have shown that these treatment options do not adequately restore meniscus hoop stress tolerance and are ineffective in preventing osteoarthritis progression [2, 3]. The primary goal of MMPRT management is to prevent osteoarthritis. Recently, meniscus preservation with meniscus root repair has become widely performed.

MMPRT repair can reduce medial joint contact pressure by enlarging the medial joint contact area [4], producing more favourable clinical outcomes than non-operative treatment [5]. Meta-analyses have shown that MMPRT repair yields good clinical outcomes and slows the progression of osteoarthritis at the mid-term timepoint, but does not prevent it completely [6]. However, the extent to which arthropathic changes progress remains controversial.

To date, osteoarthritis progression has mostly been evaluated based on the Kellgren–Lawrence (KL) classification; it has rarely been assessed using quantitative methods. Therefore, in this study, we investigated early arthropathic changes by quantitatively assessing medial joint space (MJS) widths.

The Rosenberg view is widely used to evaluate osteoarthritis and MJS. However, it is often difficult for older patients with knee pain and other symptoms to maintain an accurate Rosenberg position for a certain period, and evaluation over time is also problematic. It has been reported that radiography in a fixed-flexion view (FFV) without fluoroscopy to assist knee positioning provides accurate measurements of MJS narrowing in longitudinal studies [7, 8]. The area of cartilage degeneration after pullout repair for MMPRT is reportedly found anterior to the loading area of the medial femoral condyle [9]. In the Rosenberg view, a 45° knee flexion position is used to evaluate posterior cartilage degeneration of the medial femoral condyle. Contrastingly, in the FFV, which employs a 15–20° knee flexion position, it is possible to evaluate the anterior aspect of the medial femoral condyle. This study used the FFV to evaluate MJS narrowing widths over time based on these considerations.

The purposes of this study were to accurately assess the progression of MJS narrowing after pullout repair for MMPRT using the FFV and to identify the correlating factors associated with MJS narrowing progression. We hypothesized that after pullout repair for MMPRT, the MJS of the MMPRT knee would be narrower than that of the contralateral knee.

## Materials and methods

### Patients

**Fig. 1:**
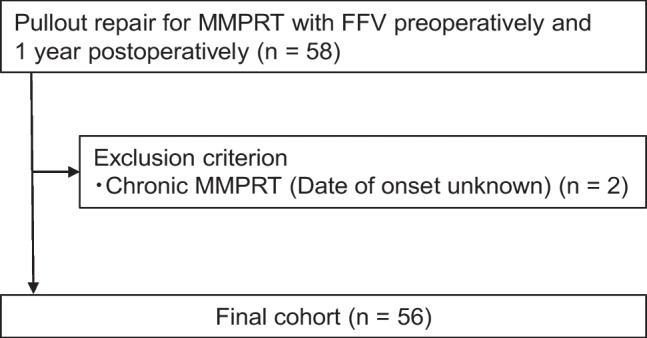
Study protocol flowchart. MMPRT, medial meniscus posterior root tear; FFV, fixed-flexion view

Our Institutional Review Board approved this retrospective study. We also obtained written informed consent from all patients. Generally, indications for pullout repair for MMPRT are a tibiofemoral angle < 180°, KL grade 0–2, and mild cartilage lesions (Outerbridge grade I or II). From June 2020 to June 2021, pre-operative and one year post-operative radiographs were taken in the FFV for 58 patients who underwent pullout repair for MMPRT. Patients in the chronic phase with unknown onset time (*n* = 2) were excluded. Finally, this study included 56 patients (Fig. 1).

The baseline characteristics (age, sex, body mass index), time from injury to surgery, surgical techniques, and clinical scores were identified for all patients (Table 1).

**Table 1:** Patient characteristics and surgical technique

Characteristics	Value
Patients, *n*	56
Age, years	64.9 ± 9.4
Sex, male/female	12/44
Body mass index, kg/m^2^	26.4 ± 4.6
MMPRT side, left/right	24/32
Time from injury to surgery, days	82.4 ± 85.5
Surgical technique, TCS/TCS + PA	38/18

Values are presented as mean ± standard deviation or number

Abbreviations: *MMPRT*, medial meniscus posterior root tear; *PA*, posterior anchoring; *TCS*, two-cinch stitches

### Radiological assessment

**Fig. 2:**
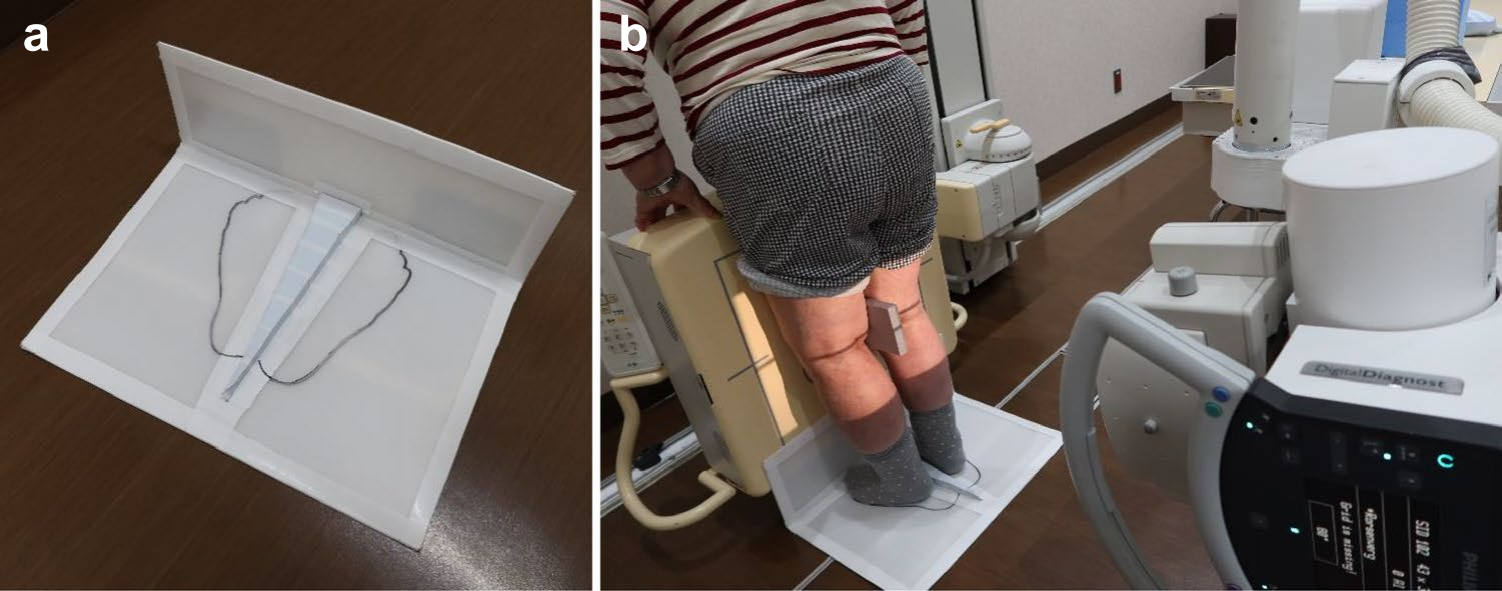
FFV methods. (**a**) The custom-made limb positioning plate. (**b**) The patient standing on the custom-made plate while keeping the toes and the front of the thighs in contact with the film cassette. A posteroanterior image is acquired with the X-ray beam angled caudally at 10°. FFV, fixed-flexion view

**Fig. 3:**
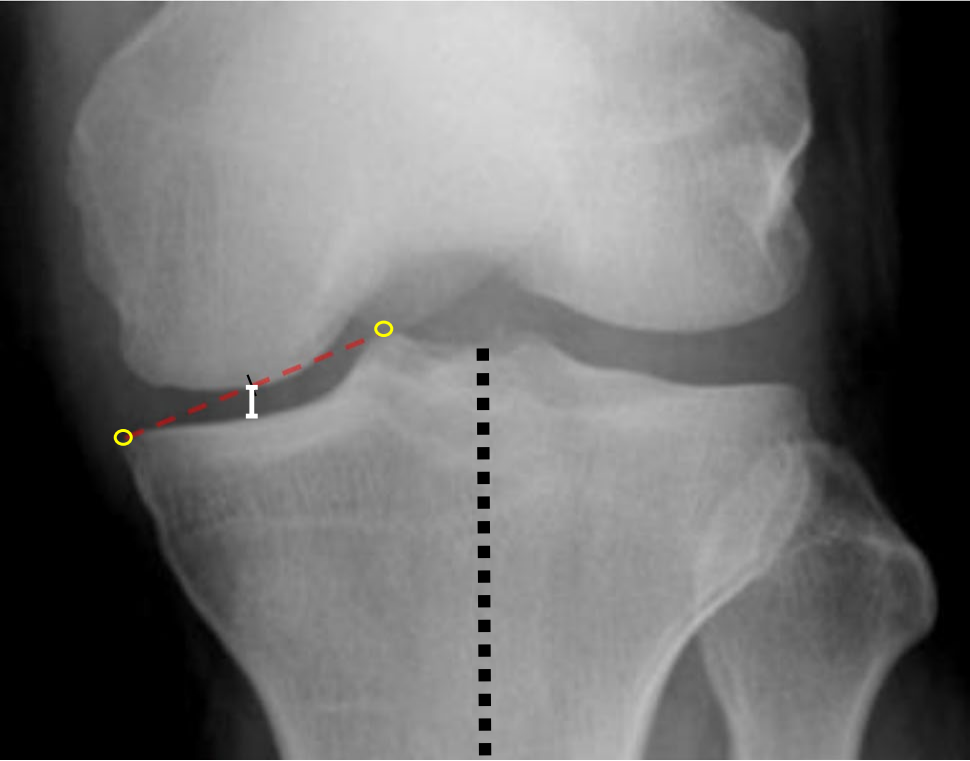
Measurement of the MJS width using the midpoint technique (left knee). The MJS (white line) is measured between the femoral and tibial surfaces at the midpoints of the medial tibial eminence and the medial point of the tibial plateau (yellow circles), parallel to the long axis of the tibia (black dotted line). MJS, medial joint space

The FFV uses an imaging limb positioning device created based on the trials of Peterfy et al. [8]. The patient stood on the custom-made plate while keeping the toes and front of the thighs in contact with the film cassette. A posteroanterior image was acquired using an X-ray beam angled caudally at 10° (Fig. 2). Radiography in the FFV was used to assess the MJS on the bilateral knees. The MJS was measured based on the midpoint technique [10, 11]. The midpoint technique measures the MJS with a line passing through the location defined by the midpoint of the medial tibial eminence and the medial margin of the medial tibial plateau, parallel to the long axis of the tibia (Fig. 3).

**Fig. 4:**
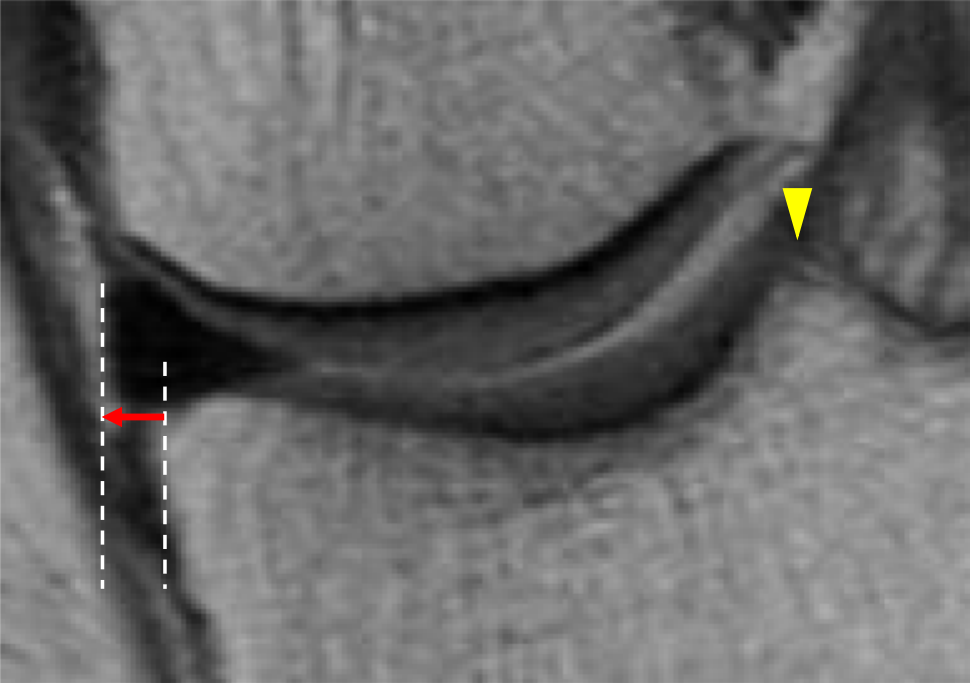
Measurement of MME using the bony landmark method (left knee). The MME is determined by measuring the horizontal distance (red arrow) between the medial point of the tibial plateau and the medial point of the meniscus in the slice where the highest medial tibial eminence (yellow arrowhead) is visible. MME, medial meniscus extrusion

Magnetic resonance imaging (MRI) was performed both pre-operatively and one year post-operatively on the MMPRT knee under an unweighted state. The MM extrusion (MME) on the MMPRT knee was measured from the medial point of the tibial plateau and the medial point of the meniscus based on the bony landmark method (Fig. 4) [12]. The increase in MME (ΔMME) was the value obtained by subtracting the post-operative MME from the pre-operative MME.

In the MJS and MME measurements, values were taken to the second decimal place.

### Clinical outcome

We assessed and compared the following scores from clinical scoring systems pre-operatively and one year post-operatively: International Knee Documentation Committee scores, pain visual analog scale scores, Knee Injury and Osteoarthritis Outcome Scores, Lysholm’s scores, and Tegner’s activity scores.

### Surgical technique and rehabilitation protocol

We performed pullout repair using the two-cinch stitches (TCS) method alone in 38 patients or using the TCS method combined with the posterior anchoring (PA) technique in 18 patients [13, 14]. The TCS method used the suture passer to thread the meniscus, and an original posterior root-aiming device to create the tibial tunnel [15]. The TCS were pulled out of the tunnel using a suture retriever. For PA, another bone tunnel was created, and all-inside sutures were inserted into the MM and bone tunnel [13].

The operated knee was constrained with a knee brace, and patients were instructed that no body weight should be supported on the knee for one week post-operatively. Thereafter, partial body weight carrying (20 kg) was allowed and gradually (+20 kg/week) transitioned to full body weight support. Knee flexion of 30° was allowed after one week and gradually (+30°/week) transitioned to 120°. Deep flexion of the knee was allowed 12 weeks post-operatively.

### Second-look arthroscopic meniscus healing scores

All patients received a second-look arthroscopy at one year post-operatively. The posterior root healing status was assessed using a previously described arthroscopic scoring system [16]. The scoring system is evaluated based on (1) anteroposterior width of bridging tissue (0−4 points), (2) stability (0–4 points), and (3) synovial coverage (0–2 points) at the posterior root of MM. The total scores are represented from 0 to 10 points. The absolute anteroposterior width of the bridging tissue was also evaluated in millimeters.

### Statistical analysis

Values are presented as mean ± standard deviation. Statistical analyses were performed using EZR (Saitama Medical Center, Saitama, Japan). A *p*-value < 0.01 was considered statistically significant. The Mann–Whitney *U* test was used to compare MJS in the MMPRT and contralateral knees. Fisher’s exact test was used to compare the KL grade in the MMPRT and contralateral knees. Wilcoxon’s signed-rank test was used to compare the pre- and post-operative results. Linear regression analysis was used to evaluate Pearson’s correlation coefficient between MJS narrowing and other factors.

MJS and MME were evaluated independently by two observers. Each observer measured the values twice, at least six weeks apart. Inter- and intraobserver reliability of the measurements was examined using the intraclass correlation coefficient (ICC). For the measurement of MJS, the ICCs ranged from 0.951 to 0.984 for intraobserver repeatability and 0.965 to 0.988 for interobserver repeatability. For the measurement of MME, the ICC was 0.868 for intraobserver repeatability and 0.847 for interobserver repeatability. All reliabilities were excellent (an ICC value of > 0.800) for each measurement.

A post hoc power analysis to identify differences in the number of MMPRT knees versus contralateral knees resulted in an actual power of 79.8% with a critical *p*-value of 0.01 (G*Power, version 3.1.9.7).

## Results

In the radiographic evaluations, the mean preoperative MJS widths of the MMPRT knees (*n* = 56) and contralateral knees (*n* = 56) were not significantly different (4.41 ± 1.04 mm vs. 4.55 ± 0.97 mm, *p* = 0.311). The mean post-operative MJS width was significantly smaller in MMPRT knees than in contralateral knees (3.90 ± 0.95 mm vs. 4.46 ± 1.03 mm, *p* = 0.003). The MJS narrowing width was significantly larger in MMPRT knees than in contralateral knees (0.51 ± 0.85 mm vs. 0.09 ± 0.49 mm, *p* < 0.001). There was no significant difference in MJS narrowing between root tear classification type 1 (*n* = 9) and type 2–4 (*n* = 47) (0.53 ± 1.04 mm vs. 0.51 ± 0.81 mm, *p* = 0.763) [17]. The pre- and post-operative KL grades were not significantly different between the MMPRT and contralateral knees, but KL grade progression in MMPRT knees was observed in 23.2% (13/56) of patients (Table 2).

**Table 2:** Comparison of MJS and KL grade between the MMPRT and contralateral knees

	MMPRT	Contralateral knee	*p*-value
	(*n* = 56)	(*n* = 56)	
Preoperative MJS, mm	4.41 ± 1.04	4.55 ± 0.97	0.311
Postoperative MJS at 1 year, mm	3.90 ± 0.95	4.46 ± 1.03	0.003*
MJS narrowing, mm	0.51 ± 0.85	0.09 ± 0.49	< 0.001*
Preoperative KL grade, 0/1/2/3/4	0/24/32/0/0	0/27/28/1/0	0.570
Postoperative KL grade at 1 year, 0/1/2/3/4	0/15/37/4/0	0/27/28/1/0	0.039

Values are presented as mean ± standard deviation

*p*-values are derived from the Mann–Whitney *U* test or Fisher’s exact test

*Statistically significant

Abbreviations: *KL*, Kellgren–Lawrence; *MJS*, medial joint space; *MMPRT*, medial meniscus posterior root tear

Regarding the MRI evaluations, the post-operative MME was significantly increased compared with the pre-operative MME (4.59 ± 1.33 mm vs. 3.43 ± 0.78 mm, *p* < 0.001).

Each clinical outcome showed significant improvement at one year post-operatively compared with the pre-operative values (*p* < 0.001) (Table 3).

**Table 3:** Comparison between pre- and post-operative clinical scores

	Preoperative (*n* = 56)	Postoperative (*n* = 56)	*p*-value
IKDC score	38.2 ± 16.8	65.8 ± 13.7	< 0.001*
VAS pain score	42.5 ± 23.4	10.3 ± 14.2	< 0.001*
KOOS			
Pain	58.2 ± 17.7	87.3 ± 11.1	< 0.001*
Symptoms	61.9 ± 20.5	80.5 ± 12.8	< 0.001*
ADL	67.7 ± 17.5	87.7 ± 10.2	< 0.001*
Sport/Rec	27.5 ± 24.0	49.0 ± 31.6	< 0.001*
QOL	37.2 ± 20.8	60.7 ± 20.6	< 0.001*
Lysholm’s score	59.9 ± 13.3	87.4 ± 7.8	< 0.001*
Tegner’s score	1.9 ± 0.8	3.1 ± 0.5	< 0.001*

Values are presented as mean ± standard deviation

*p*-values are derived from Wilcoxon’s signed-rank test

*Statistically significant

Abbreviations: *ADL*, activities of daily living; *IKDC*, International Knee Documentation Committee; *KOOS*, Knee Injury and Osteoarthritis Outcome Score; *QOL*, quality of life; *Sport/Rec*, sport and recreation function; *VAS*, visual analog scale

In the second-look arthroscopic meniscus healing scores, (1) the anteroposterior width of bridging tissue was 3.9 ± 0.4 points, (2) the stability was 2.6 ± 0.7 points, and (3) the synovial coverage was 0.9 ± 0.5 points, for a total score of 7.4 ± 1.0 points. The absolute anteroposterior width of the bridging tissue was 7.0 ± 1.5 mm. In all patients, posterior root continuity was restored.

**Fig. 5:**
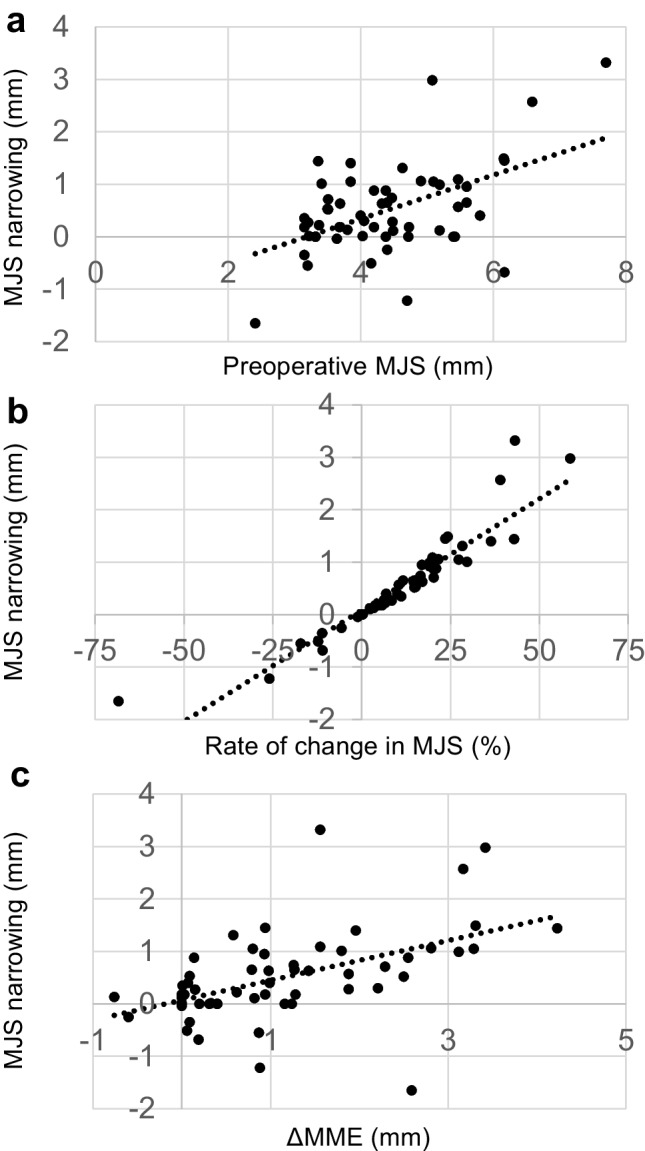
Scatter plot of the correlation between MJS narrowing and preoperative MJS (**a**), rate of change in MJS (**b**), and ΔMME (**c**). MJS, medial joint space; rate of change in MJS, medial joint space narrowing divided by preoperative medial joint space; ΔMME, the increase in medial meniscus extrusion

In Pearson’s correlation analysis, the following factors were positively correlated with MJS narrowing: pre-operative MJS (coefficient = 0.510, *p* < 0.001), rate of change in MJS (coefficient = 0.929, *p* < 0.001), and ΔMME (coefficient = 0.506, *p* < 0.001) (Table 4, Fig. 5).

**Table 4:** Pearson’s correlation between clinical variables and MJS narrowing at one year post-operatively

Variable	MJS narrowing	
	Correlation coefficient	*p*-value
Age	−0.148	0.275
Body mass index	−0.026	0.852
Sex, male/female	0.286	0.033
Time from injury to surgery	−0.243	0.071
Surgical technique, TCS/TCS + PA	0.107	0.432
Preoperative KL grade	−0.169	0.214
Preoperative MJS	0.510	< 0.001*
Preoperative MME	−0.185	0.173
ΔMME	0.506	< 0.001*
Rate of change in MJS	0.929	< 0.001*
Meniscus healing scores	0.010	0.942
Absolute anteroposterior width	0.136	0.318

*Statistically significant

Abbreviations: *ΔMME*, increase in medial meniscus extrusion; *KL*, Kellgren–Lawrence; *MJS*, medial joint space; *MME*, medial meniscus extrusion; *PA*, posterior anchoring; *Rate of change in MJS*, medial joint space narrowing divided by preoperative medial joint space; *TCS*, two-cinch sutures

## Discussion

The most important finding of this study was that the MJS narrowing width was 0.51 mm in the MMPRT knees and 0.09 mm in the contralateral knees over the first post-operative year. Quantitative measurement of MJS using the FFV showed that MMPRT knees in the early post-operative period undergo progressive osteoarthritic changes. Additionally, pullout repair for MMPRT improved clinical outcomes favorably, but it did not prevent the progression of MME and MJS narrowing.

FFV uses an imaging limb positioning device created based on the trials by Peterfy et al. [8]. The method makes it possible to easily maintain the position, despite knee pain or flexion contracture, and the work is highly reproducible. While the Rosenberg view may be superior in assessing cartilage damage and osteoarthritis at a certain time, FFV using a limb positioning device is superior in assessing the progression of joint space narrowing over time. Using the FFV allowed us to assess the progression of MJS narrowing more accurately than previously reported methods. KL grading is a widely used method of evaluating osteoarthritis via radiography. Although KL grading is an excellent evaluation method, it is not an accurate assessment method for early knee osteoarthritis. We, therefore, evaluated the MJS widths using the FFV.

The primary goal of MMPRT patient management is the prevention of osteoarthritis. One study reported that MMPRT repair inhibited the progression of osteoarthritis and resulted in good clinical outcomes within the short-term period [5]. However, there are many reports on the deterioration of KL grade after MMPRT repair [6]. Chung et al. [18] reported that 74% of MMPRT repair patients showed progression of KL grade and MJS narrowing, with a reduction of approximately 1 mm over five years. Similarly, our study showed that MMPRT is associated with the progression of MJS narrowing and does not completely prevent these changes. In our study, the MJS narrowing was 0.51 mm/year, or 0.042 mm/month, which is larger than expected. Santana et al. [19] reported that in the group that underwent meniscectomy for meniscus injury, the joint space narrowing was 0.083 mm/month for the first 12 months and 0.014 mm/month from 12 to 72 months. After the first year of pullout repair for MMPRT, MJS narrowing is expected to slow down or stop. As such, observations should be continued for several years after the first year.

There have been reports of differences in joint space narrowing due to failure of posterior root repair. Lee et al. [20] reported that the MJS narrowing of 0.16 mm in the stable healing group compared to 0.81 mm in the nonhealing group. In our study, however, there was no correlation between second-look meniscus healing scores or anteroposterior diameter of the posterior root and MJS narrowing, although the continuity of the posterior root was restored in all cases.

In our study, KL grade progression in MMPRT knees was observed in 23.2% (13/56) of patients. However, no correlation was found between the pre-operative KL grade and MJS narrowing, possibly because the KL classification cannot capture small arthropathic changes.

The only preoperative factor affecting MJS narrowing in this study was pre-operative MJS. Other pre-operative characteristics, including time from injury to surgery, and surgical techniques, had no significant effect on MJS narrowing. Moreover, the rate of change in MJS narrowing was not constant and was further positively correlated with MJS narrowing. In the long term, the MJS of the MMPRT knee may converge to similar values in many cases, regardless of the pre-operative MJS.

In patients with MMPRT, MME is a common and important finding on MRI. MME is an essential radiological parameter that reflects arthropathic changes in knee joints. After MMPRT, MME progresses rapidly within a short period [21]. There is a correlation between the degree of MME and the progression of arthropathic changes [22]. Previous studies showed that MME was not completely reduced after pullout repair of MMPRT [23]. Moon et al. [24] found that MME progressed on average from 3.6 to 5.0 mm at 33 months after pullout repair. In our study, MME was also significantly increased during the first post-operative year, and the ΔMME correlated with the progression of MJS narrowing. These results indicate that additional efforts to reduce ΔMME are required. Compared with non-operative treatment and meniscectomy, the progression of MJS narrowing and increase of the MME were slowed, but not completely prevented following MMPRT repair. However, by slowing the progression of MJS narrowing, clinical scores can be improved, and subchondral insufficiency fracture of the knee can be prevented [25].

This study has several limitations. First, the study had a short follow-up period of one year post-operatively and a relatively small sample size. A post hoc power analysis showed that the power of the test was 79.8%, which suggests that the sample size should be a little larger. Second, we evaluated the MME under an unweighted state; hence, the ΔMME might have been undervalued. Thus, a study with a larger sample size and longer follow-up period is needed to validate the present findings.

## Conclusions

The MJS narrowing width was 0.51 mm in MMPRT knees and 0.09 mm in contralateral knees over the first post-operative year. Quantitative measurement of MJS using the FFV showed that MMPRT knees exhibited progressive osteoarthritic changes. On the other hand, patients’ clinical scores improved markedly. Correlating factors for MJS narrowing were pre-operative MJS, rate of change in MJS, and ΔMME. Preventing MME progression and reducing it are essential for preventing arthropathic changes.
